# Gene correlation network analysis to identify regulatory factors in sciatic nerve injury

**DOI:** 10.1186/s13018-021-02756-0

**Published:** 2021-10-18

**Authors:** Liuxun Li, Xiaokang Du, Haiqian Ling, Yuhang Li, Xuemin Wu, Anmin Jin, Meiling Yang

**Affiliations:** 1 Department of Spine Surgery, the First Affiliated Hospital, Shenzhen University, Shenzhen Second People’s Hospital, Shenzhen, Guangdong China; 2 Department of Joint and Trauma Surgery, The Third Affiliated Hospital of Sun Yat-sen University, Guangzhou, Guangdong China; 3 Department of Endocrinology, Shenzhen Hospital of Guangzhou University of Chinese Medicine (Futian), Shenzhen, Guangdong China; 4 Department of Spine Surgery, ZhuJiang Hospital of Southern Medical University, Southern Medical University, Guangzhou, Guangdong China; 5 Department of Oncology, Shenzhen Hospital of Guangzhou University of Chinese Medicine (Futian), Shenzhen, 518034 Guangdong China

**Keywords:** Sciatic nerve injury, Weighted gene co-expression network analysis, Gene set enrichment analysis, Protein–protein interaction, Immune infiltration, Potential therapeutic agents

## Abstract

**Background:**

Sciatic nerve injury (SNI), which frequently occurs under the traumatic hip and hip fracture dislocation, induces serious complications such as motor and sensory loss, muscle atrophy, or even disabling. The present work aimed to determine the regulating factors and gene network related to the SNI pathology.

**Methods:**

Sciatic nerve injury dataset GSE18803 with 24 samples was divided into adult group and neonate group. Weighted gene co-expression network analysis (WGCNA) was carried out to identify modules associated with SNI in the two groups. Moreover, differentially expressed genes (DEGs) were determined from every group, separately. Subsequently, co-expression network and protein–protein interaction (PPI) network were overlapped to identify hub genes, while functional enrichment and Reactome analysis were used for a comprehensive analysis of potential pathways. GSE30165 was used as the test set for investigating the hub gene involvement within SNI. Gene set enrichment analysis (GSEA) was performed separately using difference between samples and gene expression level as phenotype label to further prove SNI-related signaling pathways. In addition, immune infiltration analysis was accomplished by CIBERSORT. Finally, Drug–Gene Interaction database (DGIdb) was employed for predicting the possible therapeutic agents.

**Results:**

14 SNI status modules and 97 DEGs were identified in adult group, while 15 modules and 21 DEGs in neonate group. A total of 12 hub genes was overlapping from co-expression and PPI network. After the results from both test and training sets were overlapped, we verified that the ten real hub genes showed remarkably up-regulation within SNI. According to functional enrichment of hub genes, the above genes participated in the immune effector process, inflammatory responses, the antigen processing and presentation, and the phagocytosis. GSEA also supported that gene sets with the highest significance were mostly related to the cytokine–cytokine receptor interaction. Analysis of hub genes possible related signaling pathways using gene expression level as phenotype label revealed an enrichment involved in Lysosome, Chemokine signaling pathway, and Neurotrophin signaling pathway. Immune infiltration analysis showed that Macrophages M2 and Regulatory T cells may participate in the development of SNI. At last, 25 drugs were screened from DGIdb to improve SNI treatment.

**Conclusions:**

The gene expression network is determined in the present work based on the related regulating factors within SNI, which sheds more light on SNI pathology and offers the possible biomarkers and therapeutic targets in subsequent research.

## Introduction

Sciatic nerve injuries (SNI) are one of common peripheral nerve injury (PNI), which often cause severe disability, decreased life quality, as well as tremendous social and economic burdens [1]. Sciatic nerve injuries of traumatic and iatrogenic etiologies can lead to dramatic neurological functional loss [2–4]. However, even though the best medical treatment is applied, the neurological function recovery is difficult to predict. Most existing studies have investigated the morphologies of injury and regeneration of peripheral nerves [5–7]. Nonetheless, there are still numerous barriers to be solved to attain nerve injury recovery, including target innervation specificity, low regeneration rate, target end-organ degeneration and segmental nerve defect following the extended denervation period. Consequently, to better promote the prevention and treatment of SNI, it is necessary to shed more lights on potential molecular mechanisms that regulate peripheral nerve regeneration at a broader level.

Previous research has used transcriptome analysis in rats to report that dynamic alterations in core genes and biological processes may take place within sciatic nerve stumps in the process of nerve regeneration [8, 9]. Another earlier study has used gene expression profile to explore potential hub genes and biological pathways related to the pathogenesis of SNI [10]. However, approaches adopted in such articles usually examine individual genes, whereas genes exert their functions through the co-expression gene network showing consistent biological functions in vivo. SNI is a complex pathological process, which may probably cause by multiple genes. Therefore, measuring the impacts of multiple variants genes together should be beneficial for identifying causal factors for diseases. Additionally, PNI exhibits stereotypic histopathological reactions, which indicate that the harmonious gene expression procedure is activated [11]. WGCNA is a new tool for analyzing the gene expression signature of various samples [12]. Unlike previous screening out DEGs, WGCNA clusters highly relevant genes into one module and relates it to clinical features, which may be more conductive to identify diagnostic markers and therapeutic targets [13]. To date, WGCNA has been extensively utilized in genomic research, such as glioblastoma [14], Kawasaki disease [15], schizophrenia spectrum [16], and so on. It is speculated that identification of such co-expression patterns can shed more lights on the disease-related biological pathways. Consequently, it may be of greater importance to analyze the changes in gene co-expression network during the injury and regeneration of peripheral nerves, from which to examine the molecular foundation for such morphological changes as well as modulation of local microenvironment.

**Fig. 1:**
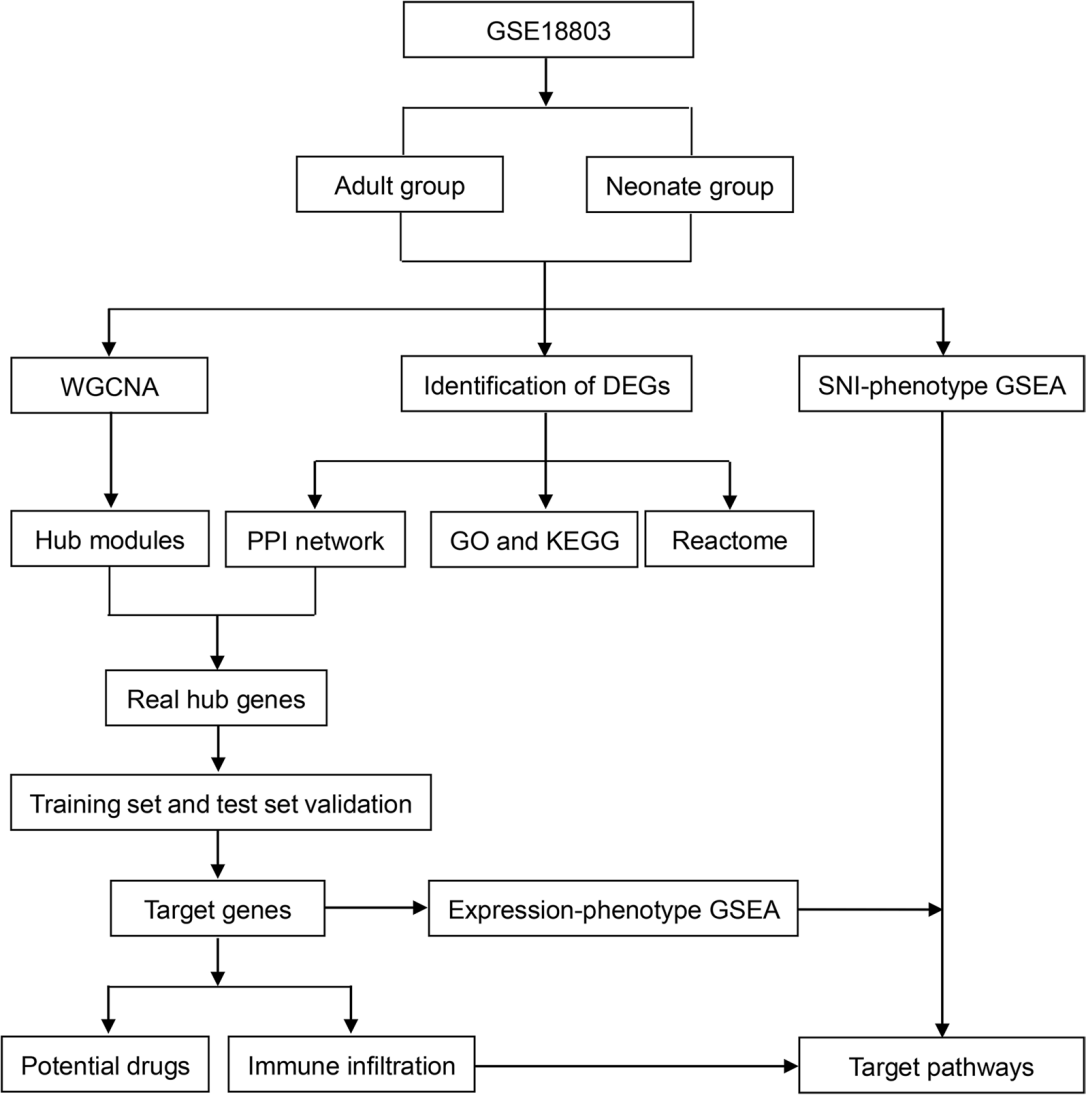
Study flow diagram

In the present study, weighted gene co-expression network analysis (WGCNA) was employed to analyze the hub genes and pathways involved in SNI pathogenic mechanism in rats of different ages. Then, the expression levels of core genes were detected using a training set and a test set, respectively. Subsequently, Reactome, Gene Ontology (GO) functional annotations, together with Kyoto Encyclopedia of Genes and Genomes (KEGG) pathway enrichment were adopted for investigating molecular mechanisms underlying SNI. To further prove SNI-related signaling pathways, GSEA was performed separately using difference between samples and gene expression level as phenotype label. Furthermore, immune infiltration analysis was carried out using CIBERSORT convolution algorithm. Finally, potential drugs or molecular compounds were predicted for improving SNI treatment by DGIdb database. These findings will help to identify the novel significant biomarkers to explore the mechanism underlying SNI development, thereby facilitating SNI diagnosis and treatment. The flow chart of the study design is presented in Fig. 1.

## Methods

### Search strategy and eligibility criteria

In the present study, we obtained the mRNA expression data of sciatic nerve injury (SNI) based on GEO database by use of keywords “sciatic nerve injury” in NCBI database (http://www.ncbi.nlm.nih.gov/geo/) until June 10, 2021. The search strategy of the study was designed as follows: (a) datasets of gene expression profiles concerning the use of the microarray chip technique; (b) studies that compared the expression data in SNI samples versus NON-SNI samples. Studies not satisfying the above-mentioned criteria were eliminated. Two reviewers searched the database independently.

### Establishment of co-expression network and analysis of module functions

Firstly, DEGs expression profiles were examined for screening the appropriate genes and samples. Secondly, the R language ‘WGCNA’ package was employed to establish the co-expression network of DEGs [17, 18]. Afterward, each pair-wise gene was functioned using Pearson’s correlation matrix. Thirdly, we adopted the power function amn = |cmn|*β* (amn represents the adjacency of gene m relative to gene *n*, whereas cmn stands for Pearson correlation of gene *m* with gene *n*) to create the weighted adjacency matrix, while *β* was the soft threshold factor adopted to stress the strong associations across genes and to penalize those weak relationships. Fourth, topological overlap matrix (TOM) adjacency was converted for measuring the gene network connectivity deemed to be the total value of the adjacency to the remaining genes in generating the network. The mean linkage hierarchical clustering was created by the dissimilarity measure based on TOM, and the minimal size (gene group) was set at 50 for gene dendrogram. Thereafter, genes that had akin expression patterns were clustered as the same gene module. Lastly, the module eigengene dissimilarity was determined. Then, such gene modules were performed functional enrichment analyses to identify the SNI-related modules. The above gene modules were conducted functional enrichment analysis for identifying related modules affecting SNI in rats of different ages.

### Identification of SNI status hub module

For identifying modules showing significant associations with illness state traits (SNI vs. NON-SNI), MEs (which represent first-principle component in one module) [12] were associated with the external traits to identify correlations with the highest significance. In addition, Gene significance (GS) determines the absolute value associations between genes and external characteristics. Meanwhile, module membership (MM) suggests the association of gene expression profiles with MEs. In this study, genes that showed the greatest GS and MM values in the interested modules were identified as the natural candidates in later analysis [19–22].

### Hub genes validation

A hub gene is substantially related to other genes within the module, which is suggested in previous studies to display functional significance. Firstly, this study screened hub genes within the co-expression network from the SNI phenotype-related module. Besides, we imported DEGs into the Search Tool for the Retrieval of Interacting Genes/Proteins (STRING) (https://string-db.org/) [23], and the confidence > 0.4 was chosen for creating the protein–protein interaction (PPI) network. Later, Cytoscape (www.cytoscape.org/) [24] was carried out for PPI network visualization. Subsequently, the Cytoscape plug-in CytoHubba was adopted for calculating every protein node degree. Nodes that had great connectivity degree were more important to maintain the network stability. Any gene within PPI network with the connectivity of ≥ 6 (node/edge) was screened to be the hub gene. Later, we deemed those shared PPI network and co-expressed hub genes as the “real” hub genes, which were selected for subsequent analysis. The Venn diagram was constructed using Venny 2.1.0 (https://bioinfogp.cnb.csic.es/tools/venny/index.html) for visualizing those common hub genes in PPI and co-expression networks between adult group and neonate group.

### Functional enrichment analyses of hub genes

GO analysis has been developed as an efficient way to carry out functional enrichment on a large scale. Besides, KEGG is also an extensively applied database that preserves excessive data on drugs, chemical substances, diseases, biological processes, and signaling pathways. In the current work, the Metascape software (http://metascape.org) [25] was employed for GO and KEGG analyses on the DEGs. *P* < 0.05 indicated statistical significance. Besides, Reactome knowledgebase (https://reactome.org/) [26–28] offers the detailed molecular data for signaling, DNA replication, transport, metabolism, along with additional cell processes as the well-organized molecular transformation network, and it represents the modified version for the classical metabolic map within the single consistent data model. In the present study, Reactome knowledgebase (https://reactome.org/) [26–28] was employed to identify 10 most significant biological functions.

### Gene set enrichment analysis

GSEA (https://software.broadinstitute.org/gsea/index.jsp) [29] has been developed as a computation approach on the basis of genesets (namely, gene groups with shared biological functions). GSEA was adopt to investigate the enrichment of previously determined biological processes within the DEG-derived gene rank. In line with gene expression levels, samples from both adult group and neonate group were separately classified as SNI samples and NON-SNI samples. To further prove the role of 10 hub genes in the development of SNI, the phenotype label was set to the expression level for each of hub genes. Metrics for ranking hub genes were calculated using Pearson’s correlation. Differential expression enrichment analysis of 10 hub genes was performed using KEGG gene sets biological process database (c2.KEGG.v4.0) from Molecular Signatures Database (MSigDB) (http://www.broad.mit.edu/gsea/msigdb/index.jsp) as a reference. Terms enriched in each gene were recognized with the thresholds for nominal *P* < 0.05 and false discovery rate (FDR) *q* < 0.25.

### Immune infiltration analysis

The CIBERSORT deconvolution algorithm was used to analyze the difference in immune infiltration between SNI and NON-SNI samples in 22 types of immune cells and immune-associated features [30]. The overlapped items that possessed the same tendency were regarded as the changes in immune characteristic. In order to further explore the effect of immune infiltration of SNI in 22 immune cells, single-sample gene set enrichment analysis (ssGSEA) was carried out in GSE18803 datasets [31]. *P* < 0.05 was considered as statistically significant.

### Identification of the potential drugs

Drug Gene Interaction Database (DGIdb) (version 4.2.0, https://www.dgidb.org) [32], one of the openly accessible database, covers information on drug–gene interaction, drug-sensitive and targeted genome. In this study, DGIdb v4.2.0 was searched for predicting the possible hub gene-interacting molecule compounds or drugs and visualizing the drug–gene interaction network through using Cytoscape.

## Results

### Included study characteristics

GSE18803 and GSE30165 microarray datasets were obtained from GEO database [9, 33]. Of them, GSE30165 dataset contained 24 samples (6 sham operation and 6 SNI samples from both neonate group and adult group) was obtained from the platform of GPL7294 Agilent-014879 Whole Rat Genome Microarray 4x44 K G4131F, whereas the GSE18803 contained microarray data acquired from ipsilateral dorsal horns at 7 days following sham operation or SNI surgery in both groups (12 from 10-day-old (neonate) group and 12 from 8–12-week-old (adult) group) based on platform GPL341 [RAE230A] Affymetrix Rat Expression 230A Array. In the present study, GSE18803 dataset was utilized to construct co-expression and PPI networks in every age group for identifying “real” hub genes and pathways. The GSE30165 microarray data was obtained from proximal sciatic nerve samples (0.5 cm) and L4–6 dorsal root ganglia samples at 0, 1, 4, 7 and 14 days following sciatic nerve removal. The GSE30165 dataset was used as the test set for result validation in the current study.

### DEGs identification

**Fig. 2:**
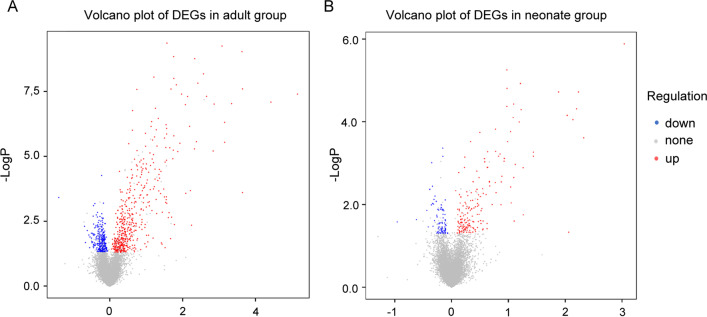
Volcano plot of DEGs. **A** Adult group. **B** Neonate group. The red nodes represent upregulated genes selected upon the |log2FC| ≥ 1.0 and *P* < 0.05 thresholds, while the blue nodes stand for downregulated genes selected upon the |log2FC| ≥ 1.0 and *P* < 0.05 thresholds, and the gray nodes indicate the nonsignificant genes

We utilized R language “limma” package to identify DEGs within the GSE18803 microarrays. DEGs were identified with the two criterions: |log2fold change (FC)|  ≥ 1 and the *P* value < 0.05. In adult group, we identified altogether 97 DEGs within SNI samples relative to normal samples, among which, 96 showed up-regulation while 1 showed down-regulation. And in neonate group, 21 DEGs were discovered to be differentially expressed between SNI and NON-SNI samples, and all these DEGs were up-regulated. Benjamini–Hochberg correction was utilized for adjusted *P* values. A volcano plot for DEGs from this microarray is shown in Fig. 2A, B.

### Weighted co-expression network construction and hub module analysis

**Fig. 3:**
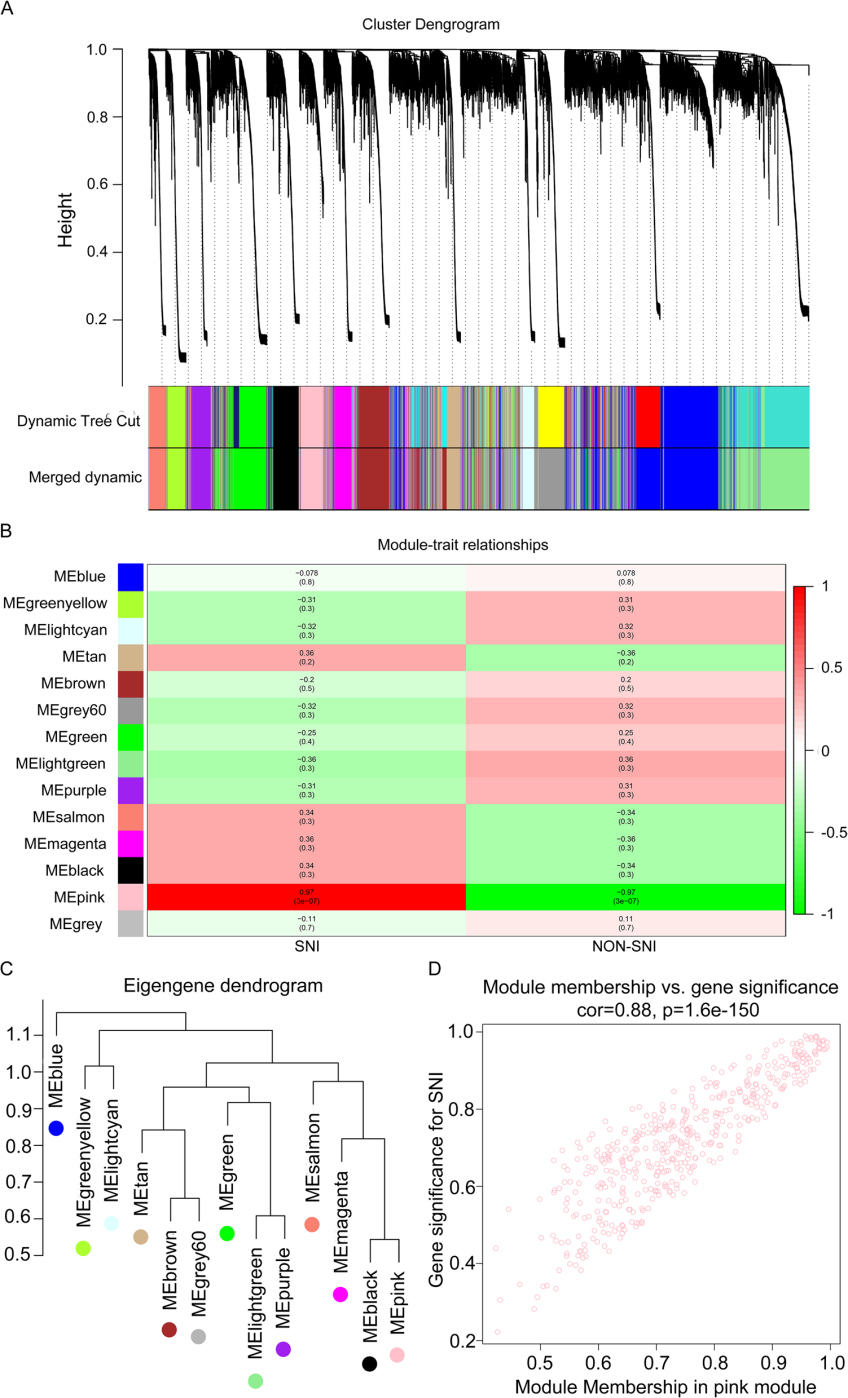
Establishment of weighted co‑expression network and analysis of hub modules in adult group. **A** Dendrogram displaying each DEG clustered in accordance with the dissimilarity measure (1-TOM). As a result, 14 co-expression modules were constructed and were shown in distinctive color. **B** Heatmap of the relationships of module with the disease traits. In the module, the greater mean gene relevance stands for the greater relationship of this module with the traits of interest. The horizontal and vertical axes stand for clinical factors and modules, respectively. The color gradient from red to green represents the shift from positive to negative correlation. The numbers in grids represent correlation coefficients. Values in parenthesis are the *P* values for the association test. **C** System clustering tree for the modules. Dendrogram showing the eigengenes in the consensus module acquired based on WGCNA regarding consensus correlations. **D** Scatter plot presenting module eigengenes within pink module

**Fig. 4:**
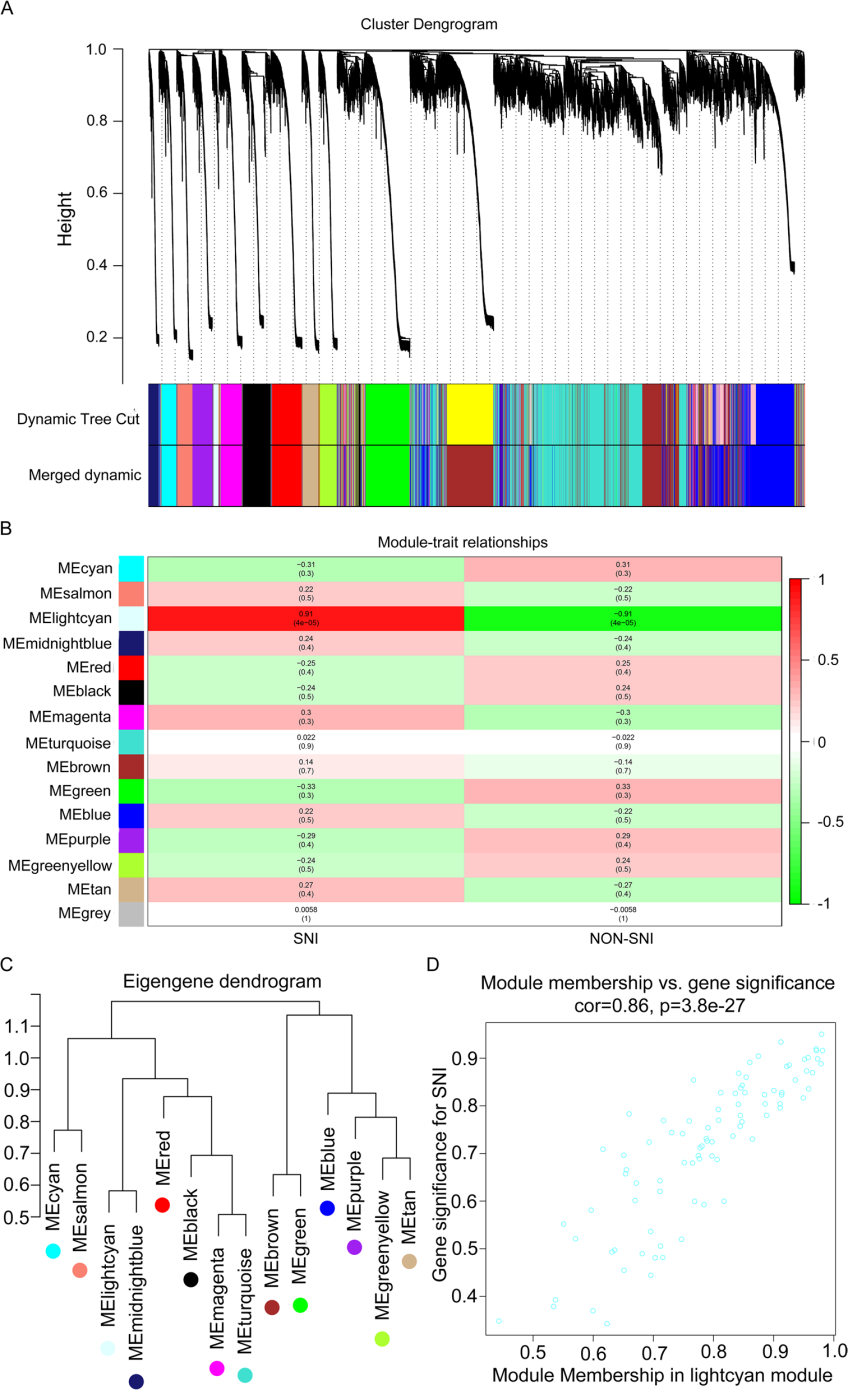
Establishment of weighted co‑expression network and analysis of hub modules in neonate group. **A** Dendrogram displaying each DEG clustered in accordance with the dissimilarity measure (1-TOM). As a result, 15 co-expression modules were constructed and were shown in distinctive color. **B** Heatmap of the relationships of module with the disease traits. In the module, the greater mean gene relevance stands for the greater relationship of this module with the traits of interest. The horizontal and vertical axes stand for clinical factors and modules, respectively. The color gradient from red to green represents the shift from positive to negative correlation. The numbers in grids represent correlation coefficients. Values in parenthesis are the *P* values for the association test. **C** System clustering tree for the modules. Dendrogram showing the eigengenes in the consensus module acquired based on WGCNA regarding consensus correlations. **D** Scatter plot presenting module eigengenes within lightcyan module

The “WGCNA” package in R language was adopted for constructing the gene co-expression networks. Then, we obtained a total 9371 genes. The 5000 most significant genes that showed the greatest standard deviations (SDs) were chosen to perform hierarchical cluster, group similar expression levels into modules, and select Power *β* = 16 to ensure a scale-free network. It was observed that 12 samples were basically classified as 2 clusters. In addition, the Pearson’s correlation was also carried out. In total, 14 modules were excavated, and the pink module was the most tightly related with SNI traits (Fig. 3A, B). Thereafter, the interactions among these 14 modules were also examined, followed by the plotting of a network heatmap. According to these findings, every module served as an independent validation for one another, demonstrating the high level of independence across various modules, as well as the relative gene expression independence for every module. For purposes of exploring co-expression similarity among these 14 modules, eigengene connectivity was assessed, and then consensus correlation was subjected to clustering analysis (Fig. 3C). In addition, intramodular analysis including MM (module significance) and GS (gene significance) was performed in those 14 modules. The pink module was excavated to further explore the highly related genes. Figure 3D displays the scatter plots for GS regarding SNI traits, together with illness state compared with MM within the pink module. In the case of SNI, GS and MM were significantly positively correlated, which suggested that the pink module elements with the greatest importance (central) might show high correlation with such external traits. Furthermore, to evaluate the influence of different ages in SNI status, the co-expression networks were similar measured using WGCNA software package in neonate group, separately. Finally, 15 modules were mined, and the lightcyan module was found to be most tightly associated with SNI traits in neonate group (Fig. 4A–D).

### Identification of SNI core genes between hub modules and PPI networks in different ages groups

**Fig. 5:**
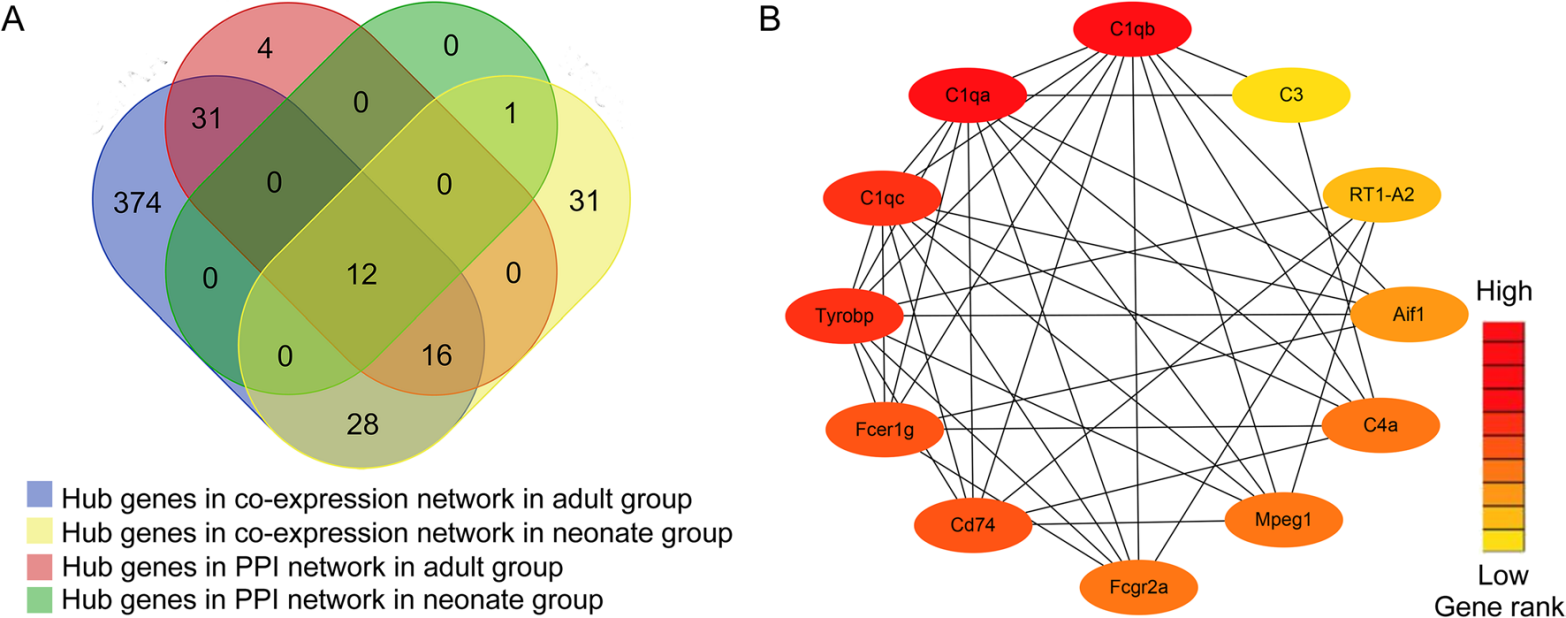
Detection of hub genes. **A** A Venn diagram presenting hub genes under co-expression and those involved in the PPI network. **B** Twelve hub genes (Cd74, C1qb, Tyrobp, C1qa, C4a, RT1-A2, C3, Fcgr2a, Aif1, Fcer1g, Mpeg1, and C1qc) overlapped between the PPI and the co-expression networks. In the heat map, intensity and color of hub genes are shown at right, which represent the gene rank 1 to 12

Subsequently, the DEGs-associated PPI network was used to identify 63 hub genes at the thresholds of connectivity ≥ 6 and confidence > 0.4 in adult group. In addition, 13 PPI networks related hub genes were screened out using similar thresholds in neonate group. The more strict factors were used in additional analyses, including module connectivity determined through absolute Pearson’s correlation coefficient (cor.geneModuleMembership > 0.8), together with relationships of clinical characteristics determined based on absolute Pearson’s correlation coefficient (cor.geneTraitSignifcance > 0.2). In adult group, there were 481 highly connected genes identified in the pink module. In contrast, 88 co-expression-related hub genes were discovered to be highly connected in lightcyan module in neonate group. Based on these analyses, 12 hub genes (C1qb, C1qa, C1qc, Tyrobp, Fcer1g, Cd74, Fcgr2a, Mpeg1, C4a, Aif1, RT1-A2, and C3) related with SNI were detected in both the co-expression and PPI networks (Fig. 5A). According to our results, each hub gene was upregulated. Therefore, the above 12 genes were identified to be real hub genes to indicate SNI status, which were screened in later analyses (Fig. 5B).

### Validation of hub genes

**Fig. 6:**
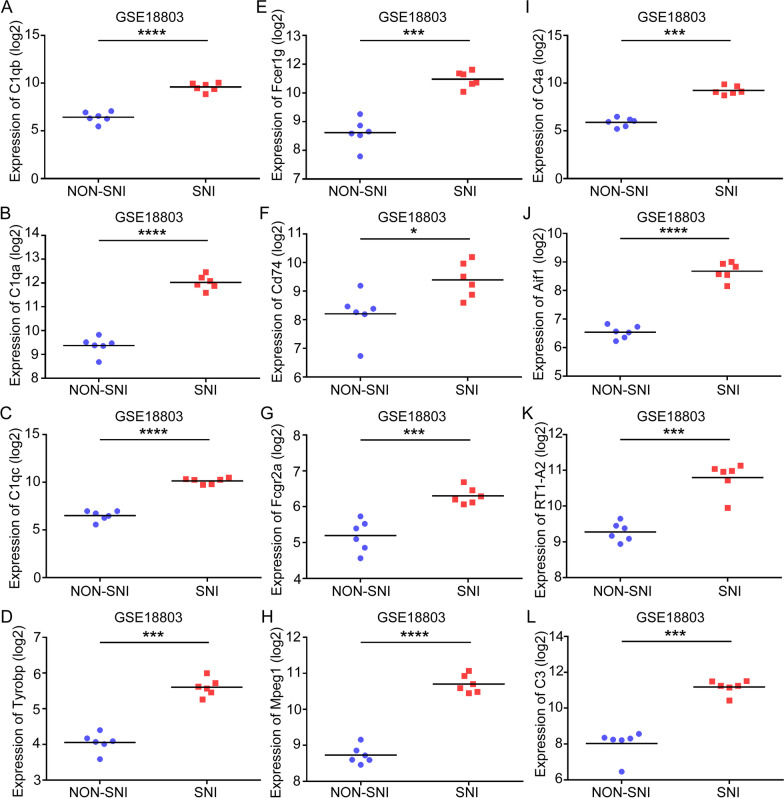
Hub gene validation based on training set (GSE18803) in adult group. The mRNA level of 12 hub genes was validated in SNI samples compared with normal samples in adult group. All hub genes revealed statistically significant differences in SNI. **A** C1qb. **B** C1qa. **C** C1qc. **D** Tyrobp. **E** Fcer1g. **F** Cd74. **G** Fcgr2a. **H** Mpeg1. **I** C4a. **J** Aif1. **K** RT1-A2. **L** C3. *P < 0.05, **P < 0.01, ***P < 0.001, *NS* not significant

**Fig. 7:**
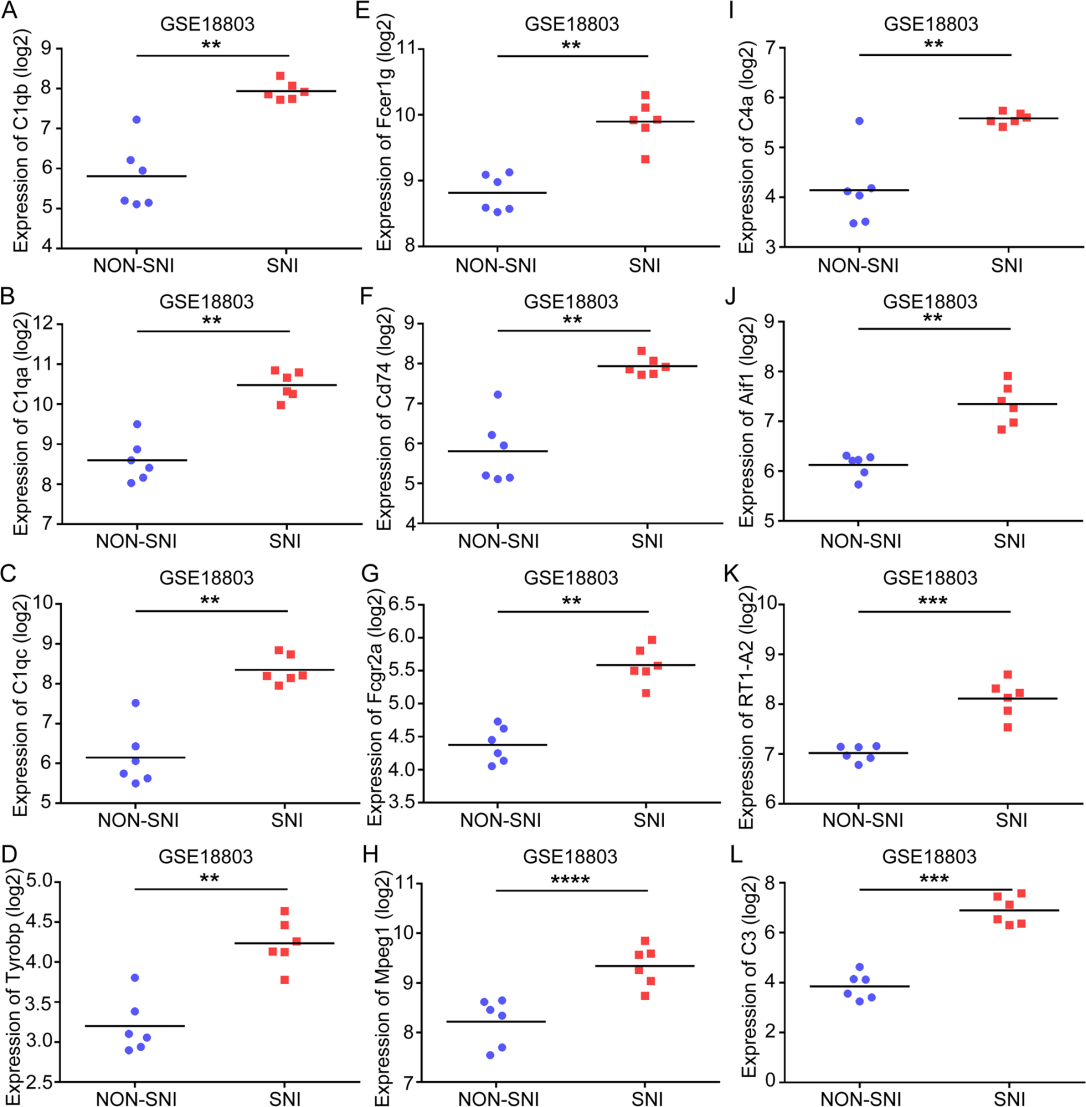
Hub gene validation based on training set (GSE18803) in neonate group. The mRNA level of 12 hub genes was validated in SNI samples compared with normal samples in neonate group. All hub genes revealed statistically significant differences in SNI. **A** C1qb. **B** C1qa. **C** C1qc. **D** Tyrobp. **E** Fcer1g. **F** Cd74. **G** Fcgr2a. **H** Mpeg1. **I** C4a. **J** Aif1. **K** RT1-A2. **L** C3. *P < 0.05, **P < 0.01, ***P < 0.001, *NS* not significant

**Fig. 8:**
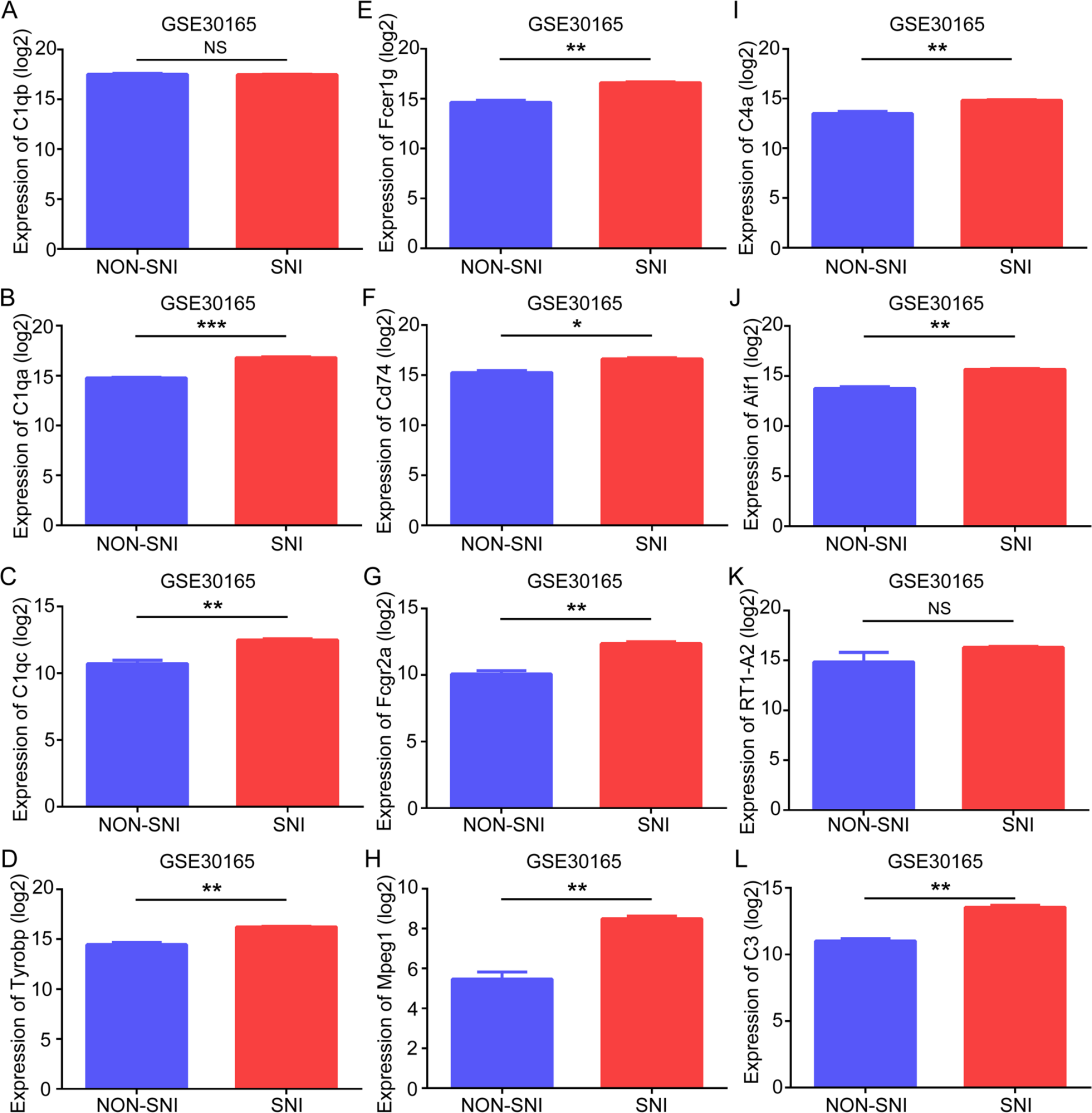
Hub gene validation based on test set (GSE30165). The mRNA level of 12 hub genes was validated in SNI samples compared with normal samples. Ten hub genes were significantly upregulated in SNI samples in comparison to NON-SNI samples. **A** C1qb. **B** C1qa. **C** C1qc. **D** Tyrobp. **E** Fcer1g. **F** Cd74. **G** Fcgr2a. **H** Mpeg1. **I** C4a. **J** Aif1. **K** RT1-A2. **L** C3. *P < 0.05, **P < 0.01, ***P < 0.001, *NS* not significant

To investigate hub genes related with SNI, the expression levels of C1qb, C1qa, C1qc, Tyrobp, Fcer1g, Cd74, Fcgr2a, Mpeg1, C4a, Aif1, RT1-A2, and C3 were detected using the training set GSE18803 dataset and the test set GSE30165 dataset, respectively. In the training set, we found all hub genes had statistically significant differences in SNI samples (Figs. 6A–I and 7A–I). In the test set, all hub genes except C1qb and RT1-A2 were significantly upregulated in the SNI samples in comparison with the NON-SNI samples (Fig. 8A–I). After overlapping the results from the training set and test set, we found ten hub genes (C1qa, C1qc, Tyrobp, Fcer1g, Cd74, Fcgr2a, Mpeg1, C4a, Aif1, and C3) were altered in the comparison between the SNI and normal control samples.

### Functional enrichment analyses of hub genes

**Fig. 9:**
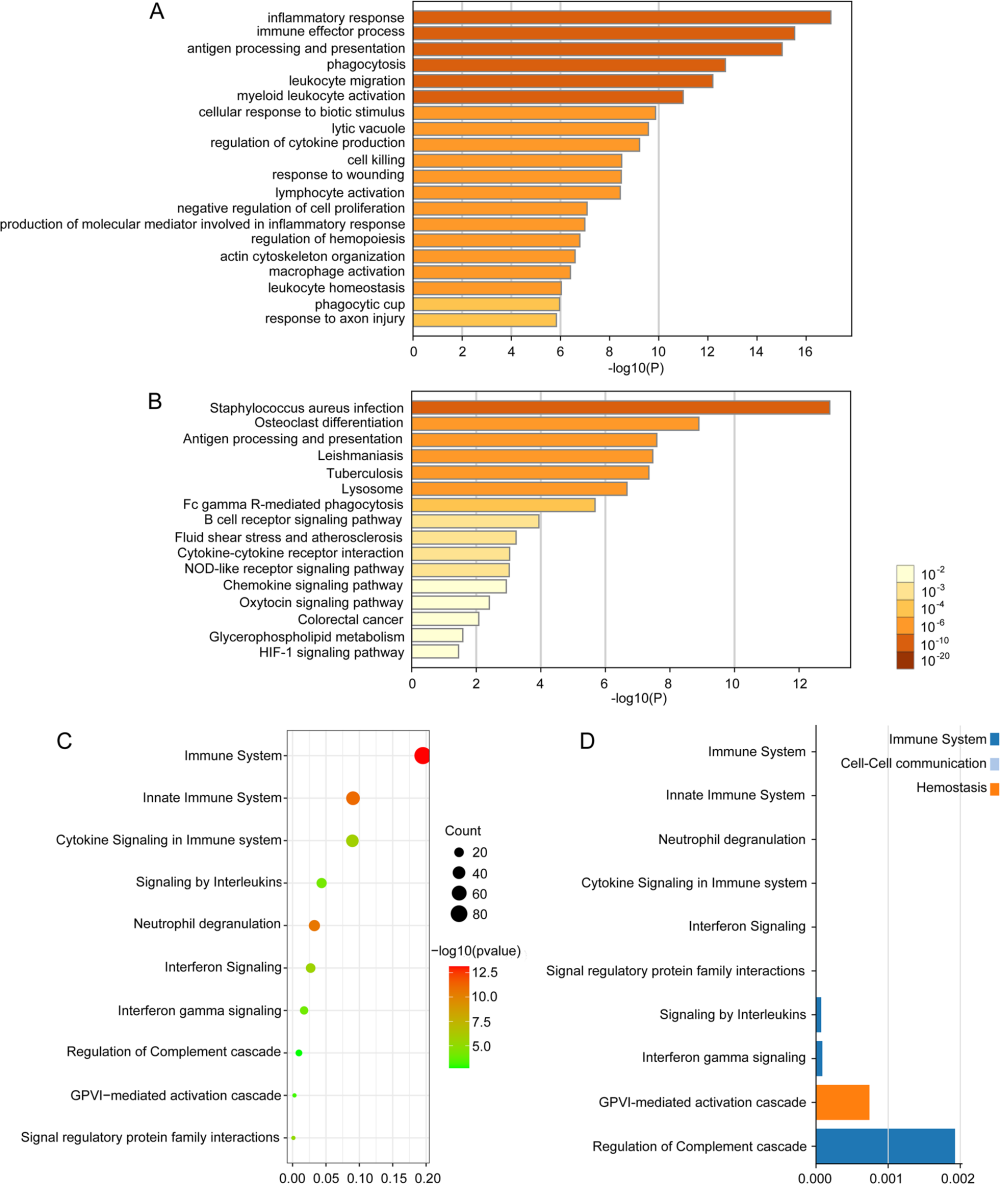
Functional enrichment analyses of hub genes. **A** Top 20 GO functional enrichment of hub genes. **B** KEGG pathways of hub genes. **C** Top 10 Reactome pathway of hub genes. The bubble pattern shows the top 10 enrichment pathways with Entities ratio, Entities found (count) and Entities FDR. *Y*-axis represents pathway name and *X*-axis represents rich factor. Size and color of each bubble represent the number of differentially expressed genes enriched in the pathway and − log10 (*q*-value), respectively. **D** Top 10 Reactome events hierarchy. The bar chart demonstrates that the gene sets involved in metabolism of proteins and signal transduction were significantly enriched in pathways related to SNI status

For better understanding gene functions in hub genes, we adopted the Metascape software for GO functional annotation and KEGG enrichment. Based on our results, “inflammatory response” was the gene set with the highest significance (Fig. 9A). The analysis also showed that SNI was associated with immune effector process, antigen processing and presentation, and phagocytosis. Meanwhile, as revealed by KEGG results, DEGs were mainly associated with antigen processing and presentation, osteoclast differentiation, and staphylococcus aureus infection pathways (Fig. 9B). For validating the biofunctions related to such hub genes, the other method was adopted. Later, Reactome, the approach for functional enrichment analysis, was applied in aligning targets with the corresponding biological functions. Thereafter, pathways were presented in the bubble chart based on the Entities found, Entities ratio, together with Entities FDR functions. As a result, the SNI samples showed remarkable relationships with immune system, innate immune system, neutrophil degranulation, and cytokine signaling in immune system (Fig. 9C). Besides, those 10 most significant functional pathways were sorted according to the entities. As observed from the bar chart, those resultant pathways exerted vital parts in immune system (Fig. 9D).

### GSEA

**Fig. 10:**
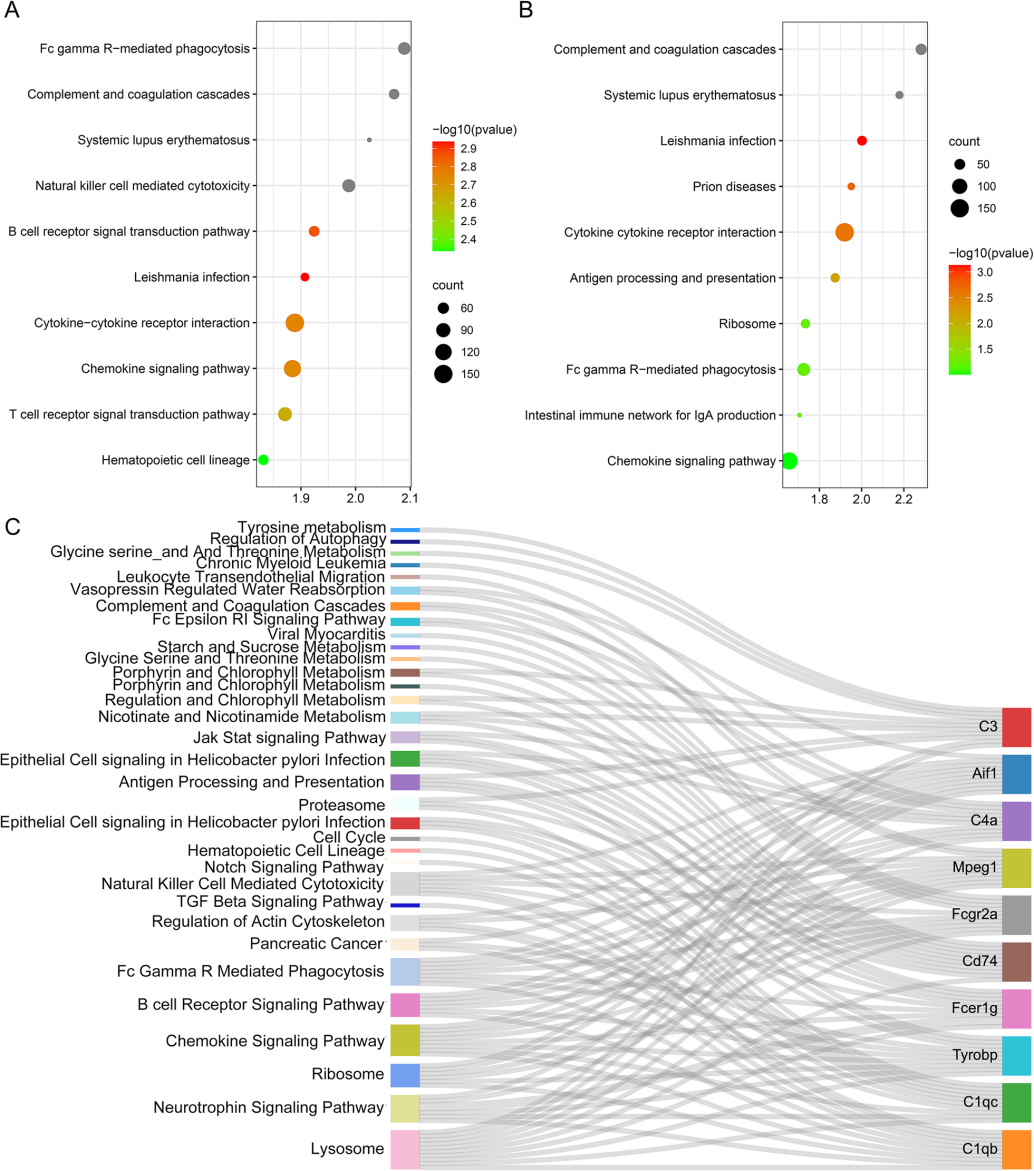
GSEA. **A** Gene set related to SNI in adult group. **B** Gene set related to SNI in neonate group. Top 10 functional gene sets enriched in SNI status between SNI samples and NON-SNI samples are shown. **C** Gene set related to each high expression in each hub genes. Sankey plot showed that high expression in each hub genes were mainly enriched in pathways associated with the Lysosome pathway, the Chemokine signaling pathway, and the Neurotrophin signaling pathway

This study conducted GSEA for identifying the potential mechanism underlying SNI. Samples were divided into SNI versus NON-SNI in both adult group and neonate group, respectively. The analysis indicated that the most significantly enriched gene sets positively correlated with SNI in adult group, which included the cytokine–cytokine receptor interaction, the chemokine signaling pathway, and the T cell receptor signal transduction pathway (Fig. 10A). In addition, significant gene sets with the highest enrichment level that showed positive correlation with SNI in neonate group were the cytokine–cytokine receptor interaction, the chemokine signaling pathway, and the Fc gamma R-mediated phagocytosis (Fig. 10B). The common pathways in both adult group and neonate group are the cytokine–cytokine receptor interaction, and the chemokine signaling pathway. To further prove the 10 hub genes-associated pathways in the development of SNI, we aligned the GEO microarray GSE18803 and focused on a single gene for the phenotype. We found that the up-regulation of 10 hub genes (C1qa, C1qc, Tyrobp, Fcer1g, Cd74, Fcgr2a, Mpeg1, C4a, Aif1, and C3) was significantly enriched in the Lysosome pathway, the Chemokine signaling pathway, and the neurotrophin signaling pathway (Fig. 10C).

### Immune infiltration analysis

**Fig. 11:**
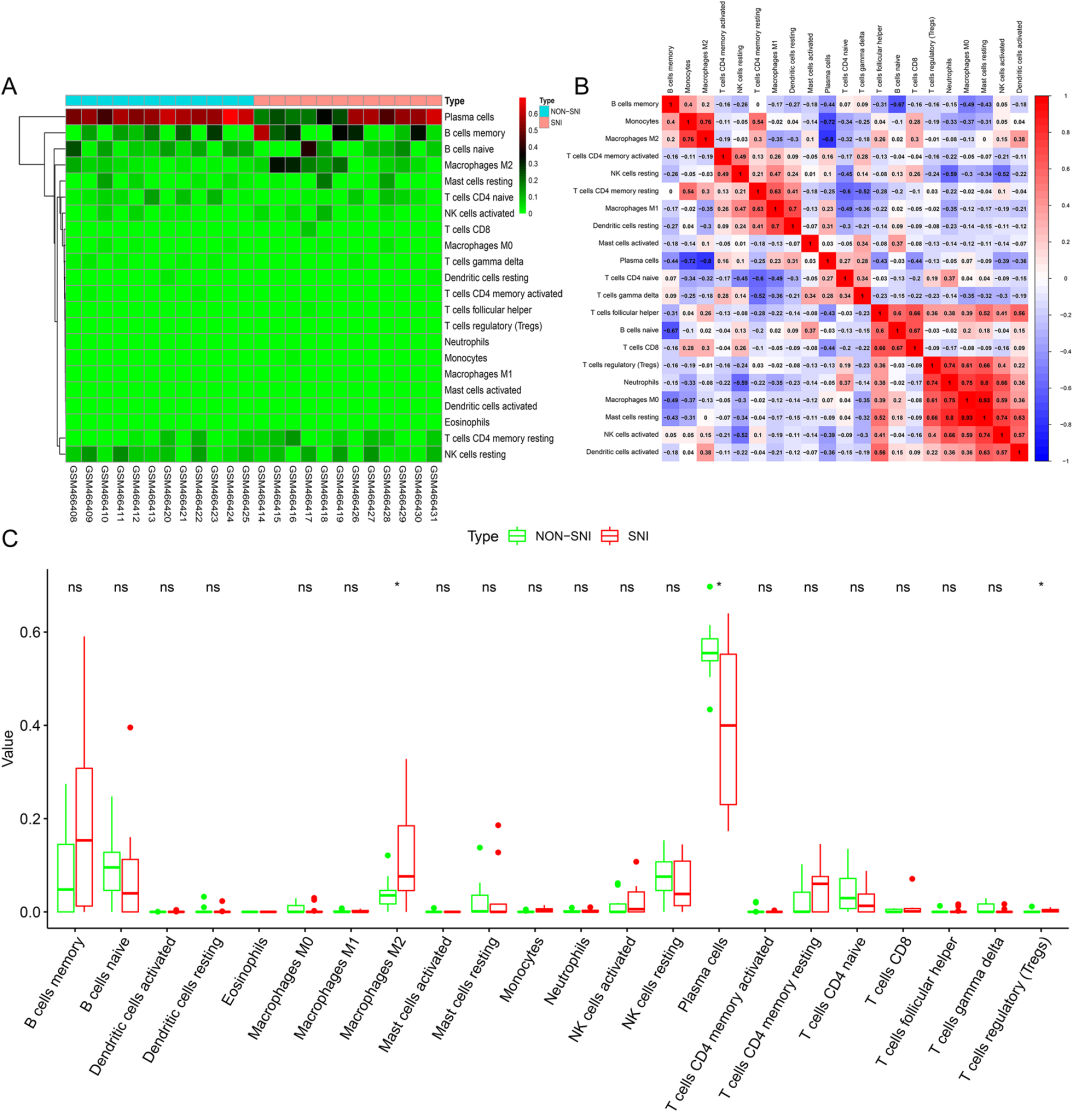
Immune infiltration analysis. **A** Heat map of the 22 immune cell proportions. **B** Correlation matrix among each type of immune cells. Red represents a positive correlation, and blue represents a negative correlation. **C** Box plots shows the differences in the proportions of the 22 immune cells between SNI samples and NON-SNI samples. Red represents SNI samples, and green represents NON-SNI samples, *P < 0.05, *NS* not significant

In order to further explore the effect of immune infiltration in SNI, the CIBERSORT deconvolution algorithm was used to analyze the difference in immune infiltration between SNI and NON-SNI samples in 22 types of immune cells. The proportion of infiltrating immune cells in each of samples was shown in Fig. 11A. In addition, a strong positive correlative links between Macrophages M0 and Mast cells activated (*R*2 = 0.93) and a clear negative correlative links between Plasma cells and Macrophages M2 (*R*2 =  − 0.80) were observed (Fig. 11B). Two immune cells (Macrophages M2 and Regulatory T cells) were found to have a statistically significant correlation with the SNI-associated risk score in the GSE18803 dataset (Fig. 11C). Therefore, these results suggested that abnormal immune infiltration may play an important role as a complex regulatory process in the progression of sciatic nerve injury and nerve regeneration. These findings may have important clinical implications of SNI.

### Identification of the potential drugs

**Fig. 12:**
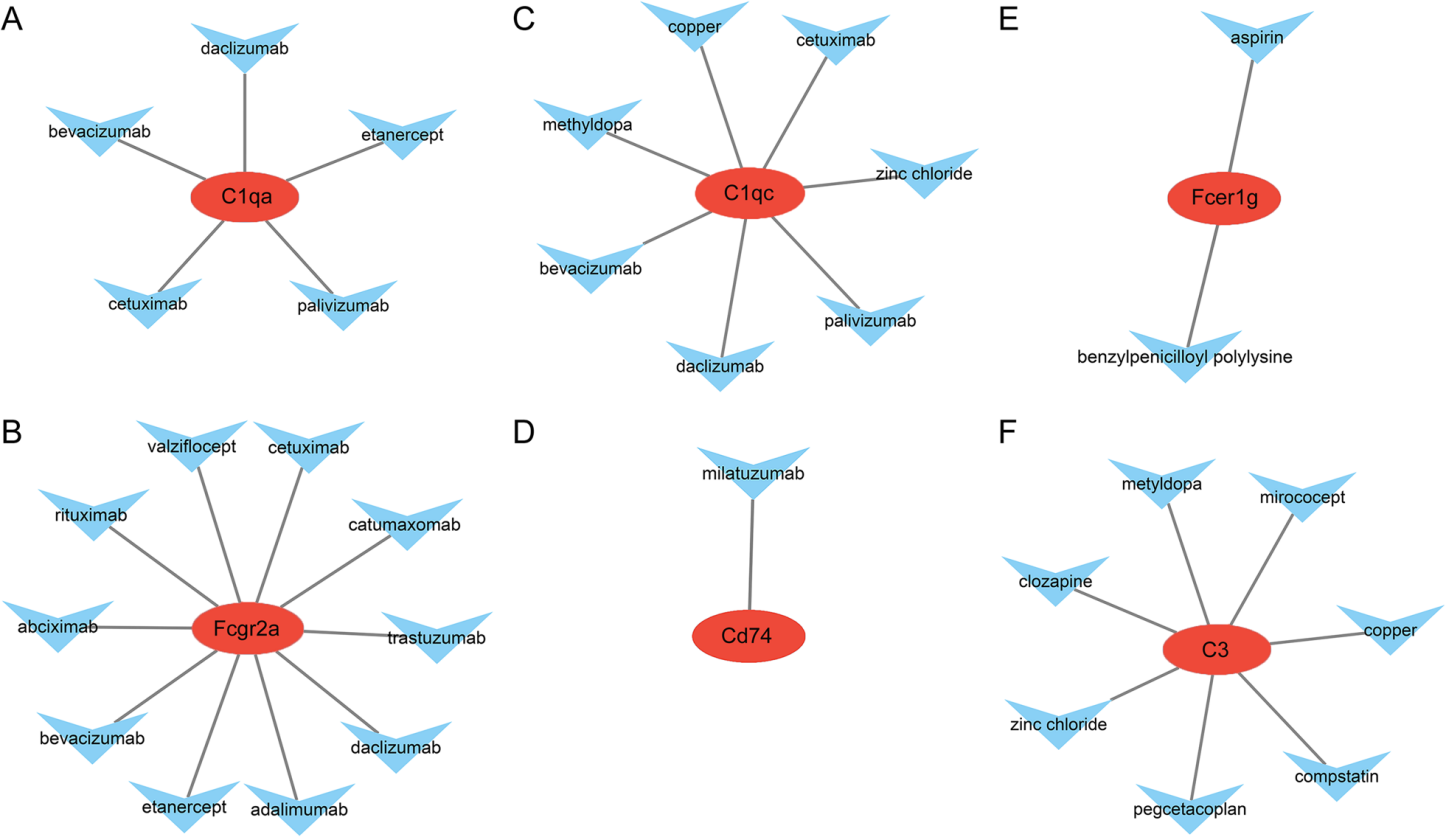
Drug–gene interaction network. The red ellipse and light-blue V nodes indicate genes and drugs, respectively. **A** C1qa. **B** Fcgr2a. **C** C1qc. **D** Cd74. **E** Fcer1g. **F** C3

This study used DGIdb for determining the possible molecular compounds or drugs with the effects on reversing the increased expression of hub genes within SNI. According to the drug–gene interaction network (Fig. 12A–F), we identified 7 molecular compounds or drugs, including metyldopa, copper and zinc chloride, and they showed differential regulation of C3 and C1QC expression. Moreover, milatuzumab was detected to interact with CD74. In addition, 10 molecular compounds or drugs, like adalimumab, adalimumab or etanercept, were identified to show interaction with FCGR2A. Furthermore, 5 molecular compounds or drugs (including etanercept) modulated C1QA, whereas 2 including aspirin and benzylpenicilloyl polylysine modulated FCER1G.

## Discussion

In the present study in which an integrated bioinformatical study on SNI was performed, an overlap method was employed to combine WGCNA, PPI network, and GSEA for identifying the hub genes as well as associated pathways. As suggested by our results, the pink module in adult group and lightcyan module in neonate group were recognized to be of clinical significance by WGCNA. In later analyses, 12 genes between co-expression and PPI networks in both adult group and neonate group were identified to be the real hub genes, which indicated the potentially vital roles of such genes during SNI occurrence and development. Subsequently, to investigate hub genes in SNI of different ages, the expression levels of the 12 genes were detected using a training set and a test set, respectively. Collectively, 10 real hub genes (C1qa, C1qc, Tyrobp, Fcer1g, Cd74, Fcgr2a, Mpeg1, C4a, Aif1, and C3) in both adult group and neonate group revealed significant differences between training set and test set.

We also conducted further potential function and pathway enrichment for clarifying the DEGs functions. According to GO analysis, DEGs related to SNI were mainly associated with inflammatory response, immune effector process, antigen processing and presentation, and phagocytosis. Consistent with KEGG enrichment analyses, the antigen processing and presentation was a significant pathway. In addition, supported in Reactome analyses, the SNI samples showed dramatically relationships with immune system, innate immune system, neutrophil degranulation, and cytokine signaling in immune system. In addition, GSEA supported that gene sets with statistical significance were mostly related to immune responses. Conforming to this work, previous studies confirmed that SNI was highly associated with inflammation [34, 35]. Immunocytes, including lymphocytes, resident cells, neutrophils, and macrophages, can produce various chemical molecules, including purines, lipids, histamine, protons, bradykinin, serotonin, chemokines, cytokines, nerve growth factors in the process of inflammation [36]. It is interesting to note that certain mediating factors show direct sensitization on nociceptors, which results in neuropathic pain [37]. These results are in line with previous studies. In the case of SNI, immune response is postponed at first, then the continuous hyperinflammatory state is detected, accompanying with the reduced repair process [38]. The inflammatory response was mostly associated with immune response usually related to lymphocytes, neutrophils and macrophages. Leukocytic infiltration may exert a certain part in catabolic enzyme generation and inflammatory response, causing the disrupted structure and function of nerve tissues. The peripheral nervous system may regrow their axons after an injury, but such capability is affected by the extracellular environment and inherent regrowing ability for supporting regrowth. Chemokines can influence neuronal differentiation, proliferation and nerve regeneration, and their expression increased in the case of inflammation and injury [39]. Nonetheless, numerous immunocytes and inflammatory factors are related to the regulation of continuous tissue damage responses, which enhances tissue repair [40]. Collectively, sciatic nerve injury and nerve regeneration display intricate biological processes, involving in the coordination of inflammatory response and immunoregulatory signals after peripheral nerve injury.

For better verifying the associations between hub genes and SNI, we obtained hub gene expression profiles based on the GEO database. The 10 genes enrolled from the above-mentioned database, including C1qa, C1qc, Tyrobp, Fcer1g, Cd74, Fcgr2a, Mpeg1, C4a, Aif1, and C3 were found to be higher in SNI as compared to the NON-SNI between adult group and neonate group. These indicated that these 10 core genes were significantly associated with SNI at both adult and neonate ages. More and more studies on transcriptomic analysis in vitro and in vivo verify that Cd74 played a vital part in the progression of sciatic nerve injury [41–44]. Linnartz-Gerlach et al. [45] reported that Tyrobp mutations or genetic variants were associated with the aging-related inflammatory neurodegenerative disorders. Another research conducted WGCNA on the expression profiles of genes specific to aging and cell-type in mice, which identified hub genes including C1qa, Tyrobp, and Fcer1g as the critical players related to neurodegenerative disorders and aging in humans [46]. According to Wang et al. [10], C1qc and Fcer1g facilitated neuropathic pain occurrence following SNI through the defense and immune pathways. C4a is related to immune responses at each level and additional events like organ regeneration and neural development [47]. Huelsenbeck et al. [48] reported that C3 peptide enhanced the functional motor recovery and axonal regeneration following PNI. As for, C1qa, C1qc, Tyrobp, Fcer1g, Fcgr2a, Mpeg1, C4a, and Aif1, they are relatively new molecules with only few reports regarding their role in SNI at present. Nevertheless, they played an important role in SNI and were significantly different between normal samples and SNI. The above genes shed more lights on clinical and experimental studies. Nonetheless, more investigation is needed to completely understand their functions in SNI. To further prove the role of hub genes in the development of SNI, differential expression enrichment analyses of the 10 hub genes were also performed. In our study, the differentially expressed genes were vast majority enriched in pathways associated with the Lysosome pathway, the Chemokine signaling pathway, and the Neurotrophin signaling pathway, which were also consistent with the results of GO and KEGG pathway enrichment analyses above.

To further explore the role of immune cell infiltration in SNI, the CIBERSORT deconvolution algorithm was used to comprehensively evaluate SNI immune infiltration. Compared with the NON-SNI samples, macrophages M2 and regulatory T cells were increased in the SNI samples, while plasma cells were decreased. Previous studies have found that M2 macrophages and regulatory T cells can promote the progression of SNI and activate the immune responses in the injured tissue [49–51] in a rodent model of sciatic nerve injury, which is consistent with our findings. To the best of our knowledge, there is no previous study about the association between plasma cells and SNI. Therefore, the relationship among M2 macrophages, regulatory T cells, and plasma cells remains to be further studied.

For predicting the candidate efficient treatment against SNI and the associated concurrent diseases, this study used DGIdb database for determining the therapeutic agents showing effects on reversing the abnormal up-regulation of SNI-associated hub genes. Tumor necrosis factor-alpha (TNF-α) is suggested to exert a vital part during demyelination and apoptosis, while blocking its expression enhances neural healing [52]. According to previous reports, TNF-α antagonists are effective on Schwann cells and axons within SNI, and TNF-α is related to the modulation of axonal regeneration [53]. An increasing number of epidemiological studies have suggested that, the anti-TNF-α therapies, including adalimumab, etanercept and adalimumab, are utilized to treat different peripheral nerve diseases, including chronic inflammatory demyelinating polyneuropathy, Miller Fisher syndrome, Guillain–Barré syndrome, mononeuropathy multiplex, multifocal motor neuropathy accompanied by conduction block, and axonal sensorimotor polyneuropathy [54]. Adalimumab was detected as the efficient neuroprotective drug to heal the nerves in PNI model of rats, particularly in the early phase [52]. Trastuzumab have also been reported to enhance peripheral nerve regeneration following repair from acute and chronic PNI [55, 56]. More studies are needed to explore the functions of the above-mentioned molecular compounds and drugs within SNI, together with the corresponding concurrent diseases as the candidate therapeutic targets.

Nonetheless, certain limitations should still be noted. First, this work identified numerous new pathways related to SNI, but it was still restricted due to the intrinsic biases of enrichment analysis and the microarray data available. Second, this study obtained the open-sourced data, but data quality was not assessed; besides, it adopted the uncommonly utilized Affymetrix gene expression arrays. Third, Mus musculus-derived tissue samples of training set were different from the Rattus norvegicus-derived samples of test set, and this might lead to diverse target genes in the 2 organisms following nerve injury. Therefore, if the database has samples updates, more studies are warranted in the future. Fourth, laboratory experiments should be carried out to verify our results. Cells isolated from SNI samples needed to be cultured in vitro for determining the related molecular mechanisms of hub gene expression. Thus, the gene knockdown preclinical animal models can help to examine the identified gene functions and evaluate their functions in SNI. Fifth, to increase the result reliability, we need more samples for repeated measurements. Last, enrichment analysis was also limited in identifying pathways because the gene lists verified might lead to over-representation of the well-identified pathways. As a result, the functions of such hub genes as well as pathways within SNI, and the functional meaning in SNI development should be validated in more research.

## Conclusion

To sum up, through a series of integrated bioinformatics analyses, we screened a total of 10 hub genes with verified high expression within SNI, and predicted potential therapeutic agents associated with the progression of SNI. According to our findings, some pathways related to SNI conformed to the known knowledge regarding disease pathology. Results in this study can shed more lights on biological pathways related to SNI and identify some possible regulating factors as the interventional targets. Nonetheless, more research is required to verify the association of hub gene functions with the immune responses during SNI development.

## Data Availability

All supporting data can be provided upon request to the authors.

## Abbreviations

SNI: Sciatic nerve injury
WGCNA: Weighted gene co-expression network analysis
DEG: Differentially expressed gene
PPI: Protein–protein interaction
GESA: Gene set enrichment analysis
DGIdb: Drug Gene Interaction Database
PNI: Peripheral nerve injury
GO: Gene Ontology
KEGG: Kyoto Encyclopedia of Genes and Genomes
GEO: Gene Expression Omnibus
TOM: Topological overlap matrix
GS: Gene significance
MM: Module membership
STRING: Search Tool for the Retrieval of Interacting Genes
FDR: False discovery rate
C1qb: Complement C1q B chain
C1qa: Complement C1q A chain
C1qc: Complement C1q subcomponent subunit C
Tyrobp: Transmembrane immune signaling adaptor TYROBP
Fcer1g: Fc fragment Of IgE receptor Ig
Cd74: CD74 molecule
Fcgr2a: Fc fragment of IgG receptor IIa
Mpeg1: Macrophage expressed gene 1
C4a: Complement C4A
Aif1: Allograft inflammatory factor 1
RT1-A2: RT1 class I, A2
C3: Complement C3
TNF-α: Tumor necrosis factor-alpha

## References

[1] De la Rosa MB, Kozik EM, Sakaguchi DS (2018). Adult stem cell-based strategies for peripheral nerve regeneration. Adv Exp Med Biol.

[2] Neer CS, Grantham SA, Foster RR (1970). Femoral shaft fracture with sciatic nerve palsy. JAMA.

[3] Aufranc OE, Jones WN, Turner RH, Thomas WH (1967). Fracture of acetabulum with dislocation of hip and sciatic palsy. JAMA.

[4] Jolles BM, Bogoch ER (2006). Posterior versus lateral surgical approach for total hip arthroplasty in adults with osteoarthritis. Cochrane Database Syst Rev.

[5] Tallon C, Rockenstein E, Masliah E, Farah MH (2017). Increased BACE1 activity inhibits peripheral nerve regeneration after injury. Neurobiol Disease..

[6] Ertürk A, Hellal F, Enes J, Bradke F (2007). Disorganized microtubules underlie the formation of retraction bulbs and the failure of axonal regeneration. J Neurosci.

[7] Qu WR, Zhu Z, Liu J, Song DB, Tian H, Chen BP (2021). Interaction between Schwann cells and other cells during repair of peripheral nerve injury. Neural Regen Res.

[8] Zhao H, Duan LJ, Sun QL, Gao YS, Yang YD, Tang XS (2020). Identification of key pathways and genes in L4 dorsal root ganglion (DRG) after sciatic nerve injury via microarray analysis. J Investig Surg Off J Acad Surg Res.

[9] Li S, Liu Q, Wang Y, Gu Y, Liu D, Wang C (2013). Differential gene expression profiling and biological process analysis in proximal nerve segments after sciatic nerve transection. PLoS ONE.

[10] Wang J, Ma SH, Tao R, Xia LJ, Liu L, Jiang YH (2017). Gene expression profile changes in rat dorsal horn after sciatic nerve injury. Neurol Res.

[11] Bosse F, Hasenpusch-Theil K, Küry P, Müller HW (2006). Gene expression profiling reveals that peripheral nerve regeneration is a consequence of both novel injury-dependent and reactivated developmental processes. J Neurochem.

[12] Langfelder P, Horvath S (2008). WGCNA: an R package for weighted correlation network analysis. BMC Bioinform.

[13] Zhang H, Guo L, Zhang Z, Sun Y, Kang H, Song C (2019). Co-expression network analysis identified gene signatures in osteosarcoma as a predictive tool for lung metastasis and survival. J Cancer.

[14] Zhou J, Guo H, Liu L, Hao S, Guo Z, Zhang F (2021). Construction of co-expression modules related to survival by WGCNA and identification of potential prognostic biomarkers in glioblastoma. J Cell Mol Med.

[15] Esmaeili S, Mehrgou A, Kakavandi N, Rahmati Y (2020). Exploring Kawasaki disease-specific hub genes revealing a striking similarity of expression profile to bacterial infections using weighted gene co-expression network analysis (WGCNA) and co-expression modules identification tool (CEMiTool): an integrated bioinformatics and experimental study. Immunobiology.

[16] Feltrin AS, Tahira AC, Simões SN, Brentani H, Martins DC (2019). Assessment of complementarity of WGCNA and NERI results for identification of modules associated to schizophrenia spectrum disorders. PLoS ONE.

[17] Mason MJ, Fan G, Plath K, Zhou Q, Horvath S (2009). Signed weighted gene co-expression network analysis of transcriptional regulation in murine embryonic stem cells. BMC Genomics.

[18] Horvath S, Dong J (2008). Geometric interpretation of gene coexpression network analysis. PLoS Comput Biol.

[19] Horvath S, Zhang B, Carlson M, Lu KV, Zhu S, Felciano RM (2006). Analysis of oncogenic signaling networks in glioblastoma identifies ASPM as a molecular target. Proc Natl Acad Sci U S A.

[20] Ghazalpour A, Doss S, Zhang B, Wang S, Plaisier C, Castellanos R (2006). Integrating genetic and network analysis to characterize genes related to mouse weight. PLoS Genet.

[21] Fuller TF, Ghazalpour A, Aten JE, Drake TA, Lusis AJ, Horvath S (2007). Weighted gene coexpression network analysis strategies applied to mouse weight. Mamm Genome Off J Int Mamm Genome Soc.

[22] Oldham MC, Horvath S, Geschwind DH (2006). Conservation and evolution of gene coexpression networks in human and chimpanzee brains. Proc Natl Acad Sci U S A.

[23] Szklarczyk D, Franceschini A, Wyder S, Forslund K, Heller D, Huerta-Cepas J (2015). STRING v10: protein–protein interaction networks, integrated over the tree of life. Nucleic Acids Res.

[24] Shannon P, Markiel A, Ozier O, Baliga NS, Wang JT, Ramage D (2003). Cytoscape: a software environment for integrated models of biomolecular interaction networks. Genome Res.

[25] Zhou Y, Zhou B, Pache L, Chang M, Khodabakhshi AH, Tanaseichuk O (2019). Metascape provides a biologist-oriented resource for the analysis of systems-level datasets. Nat Commun.

[26] Jassal B, Matthews L, Viteri G, Gong C, Lorente P, Fabregat A (2020). The reactome pathway knowledgebase. Nucleic Acids Res.

[27] Fabregat A, Sidiropoulos K, Viteri G, Marin-Garcia P, Ping P, Stein L (2018). Reactome diagram viewer: data structures and strategies to boost performance. Bioinformatics (Oxford, England).

[28] Sidiropoulos K, Viteri G, Sevilla C, Jupe S, Webber M, Orlic-Milacic M (2017). Reactome enhanced pathway visualization. Bioinformatics (Oxford, England)..

[29] Subramanian A, Kuehn H, Gould J, Tamayo P, Mesirov JP (2007). GSEA-P: a desktop application for gene set enrichment analysis. Bioinformatics (Oxford, England)..

[30] Newman AM, Liu CL, Green MR, Gentles AJ, Feng W, Xu Y (2015). Robust enumeration of cell subsets from tissue expression profiles. Nat Methods.

[31] Barbie DA, Tamayo P, Boehm JS, Kim SY, Moody SE, Dunn IF (2009). Systematic RNA interference reveals that oncogenic KRAS-driven cancers require TBK1. Nature.

[32] Cotto KC, Wagner AH, Feng YY, Kiwala S, Coffman AC, Spies G (2018). DGIdb 3.0: a redesign and expansion of the drug–gene interaction database. Nucleic Acids Res.

[33] Costigan M, Moss A, Latremoliere A, Johnston C, Verma-Gandhu M, Herbert TA (2009). T-cell infiltration and signaling in the adult dorsal spinal cord is a major contributor to neuropathic pain-like hypersensitivity. J Neurosci Off J Soc Neurosci.

[34] Huang TC, Wu HL, Chen SH, Wang YT, Wu CC (2020). Thrombomodulin facilitates peripheral nerve regeneration through regulating M1/M2 switching. J Neuroinflamm.

[35] Neumann E, Küpfer L, Zeilhofer HU (2020). The α2/α3GABAA receptor modulator TPA023B alleviates not only the sensory but also the tonic affective component of chronic pain in mice. Pain.

[36] Finnerup NB, Kuner R, Jensen TS (2020). Neuropathic pain: from mechanisms to treatment. Physiol Rev.

[37] Shutov LP, Warwick CA, Shi X, Gnanasekaran A, Shepherd AJ, Mohapatra DP (2016). The complement system component C5a produces thermal hyperalgesia via macrophage-to-nociceptor signaling that requires NGF and TRPV1. J Neurosci Off J Soc Neurosci.

[38] Büttner R, Schulz A, Reuter M, Akula AK, Mindos T, Carlstedt A (2018). Inflammaging impairs peripheral nerve maintenance and regeneration. Aging Cell.

[39] Deftu AT, Ciorescu R, Gheorghe RO, Mihăilescu D, Ristoiu V (2019). CXCL1 and CXCL2 inhibit the axon outgrowth in a time- and cell-type-dependent manner in adult rat dorsal root ganglia neurons. Neurochem Res.

[40] Jiang BC, Liu T, Gao YJ (2020). Chemokines in chronic pain: cellular and molecular mechanisms and therapeutic potential. Pharmacol Ther.

[41] Song H, Zhu Z, Zhou Y, Du N, Song T, Liang H (2019). MIF/CD74 axis participates in inflammatory activation of Schwann cells following sciatic nerve injury. J Mol Histol.

[42] Sun W, Kou D, Yu Z, Yang S, Jiang C, Xiong D (2020). A transcriptomic analysis of neuropathic pain in rat dorsal root ganglia following peripheral nerve injury. Neuromol Med.

[43] Yang JA, He JM, Lu JM, Jie LJ (2018). Jun, Gal, Cd74, and C1qb as potential indicator for neuropathic pain. J Cell Biochem.

[44] Wang F, Xu S, Shen X, Guo X, Peng Y, Yang J (2011). Spinal macrophage migration inhibitory factor is a major contributor to rodent neuropathic pain-like hypersensitivity. Anesthesiology.

[45] Linnartz-Gerlach B, Bodea LG, Klaus C, Ginolhac A, Halder R, Sinkkonen L (2019). TREM2 triggers microglial density and age-related neuronal loss. Glia.

[46] Mukherjee S, Klaus C, Pricop-Jeckstadt M, Miller JA, Struebing FL (2019). A microglial signature directing human aging and neurodegeneration-related gene networks. Front Neurosci.

[47] Klos A, Wende E, Wareham KJ, Monk PN (2013). International Union of Basic and Clinical Pharmacology. [Corrected]. LXXXVII. Complement peptide C5a, C4a, and C3a receptors. Pharmacol Rev.

[48] Huelsenbeck SC, Rohrbeck A, Handreck A, Hellmich G, Kiaei E, Roettinger I (2012). C3 peptide promotes axonal regeneration and functional motor recovery after peripheral nerve injury. Neurother J Am Soc Exp NeuroTher.

[49] Lin T, Liu S, Chen S, Qiu S, Rao Z, Liu J (2018). Hydrogel derived from porcine decellularized nerve tissue as a promising biomaterial for repairing peripheral nerve defects. Acta Biomater.

[50] Li Y, Yao D, Zhang J, Liu B, Zhang L, Feng H (2017). The effects of epidermal neural crest stem cells on local inflammation microenvironment in the defected sciatic nerve of rats. Front Mol Neurosci.

[51] Lees JG, Duffy SS, Perera CJ, Moalem-Taylor G (2015). Depletion of Foxp3+ regulatory T cells increases severity of mechanical allodynia and significantly alters systemic cytokine levels following peripheral nerve injury. Cytokine.

[52] Polat E, Dağlıoğlu E, Menekşe G, Dike MS, Özdöl Ç, Türk C (2016). Neuroprotective effects of adalimumab on rats with experimental peripheral nerve injury: an electron microscopic and biochemical study. Ulusal travma ve acil cerrahi dergisi = Turk J Trauma Emerg Surg TJTES.

[53] Smith D, Tweed C, Fernyhough P, Glazner GW (2009). Nuclear factor-kappaB activation in axons and Schwann cells in experimental sciatic nerve injury and its role in modulating axon regeneration: studies with etanercept. J Neuropathol Exp Neurol.

[54] Stübgen JP (2008). Tumor necrosis factor-alpha antagonists and neuropathy. Muscle Nerve.

[55] Hendry JM, Alvarez-Veronesi MC, Placheta E, Zhang JJ, Gordon T, Borschel GH (2016). ErbB2 blockade with Herceptin (trastuzumab) enhances peripheral nerve regeneration after repair of acute or chronic peripheral nerve injury. Ann Neurol.

[56] Placheta E, Hendry JM, Wood MD, Lafontaine CW, Liu EH, Cecilia Alvarez Veronesi M (2014). The ErbB2 inhibitor Herceptin (Trastuzumab) promotes axonal outgrowth 4 weeks after acute nerve transection and repair. Neurosci Lett.

